# Gross Anatomy Teaching for Medical Undergraduates Through Computer-Based Simulation: Introduction and Evaluation of Effectiveness

**DOI:** 10.7759/cureus.49517

**Published:** 2023-11-27

**Authors:** Navbir Pasricha, Dinesh K Badyal, Parmod Kumar Goyal, Eti Sthapak

**Affiliations:** 1 Anatomy, Dr. Ram Manohar Lohia Institute of Medical Sciences, Lucknow, IND; 2 Pharmacology, Christian Medical College and Hospital, Ludhiana, IND; 3 Forensic Medicine and Toxicology, Adesh Institute of Medical Sciences and Research, Bathinda, IND

**Keywords:** problem-based learning (pbl), virtual dissection, anatomy teaching and learning, 3-d visualization, cadaveric dissection, computer simulation dissection

## Abstract

Background

Cadaveric teaching has been the gold standard for gross anatomy instruction through the ages and across the geographic spectrum, but with issues of availability faced in many medical schools, there is a need to look for other options. Digital tools like virtual dissectors that simulate the cadaver have been around for some years now, but their acceptability to the teachers and students and effectiveness need to be validated in the settings where applied.

Aim

To evaluate the acceptability, feasibility and effectiveness of using computer-based simulation tools for teaching gross anatomy via online mode to undergraduate medical students.

Methodology

A prospective crossover randomized controlled study was conducted online on 200 (120 males (60%) and 80 females (40%), Year 1 medical undergraduates (mean age males: 19.67 years and females: 19.52 years), wherein two broad topics of head and neck region were taught by didactic lectures delivered online via Zoom. Dissection videos were prepared for both cadaveric and computer-based simulation teaching. Groups were divided by random allocation and pre- and post-tests and feedback surveys were conducted online.

Results

A significant increase from pre- to post-test scores was found in both cadaveric and computer-based simulation techniques. However, more change was found in the computer technique as its t-value was more than the cadaveric technique. The feedback from the students was that the computer-based simulation teaching method gave them a good insight into 3D understanding of the human body, increased understanding of relations of body structures and capacity to grasp surface anatomy.

Conclusion

The study concluded that teaching gross anatomy through computer-based simulation techniques is acceptable to both the students and faculty. The study also concluded that it is an effective and feasible method that can be used to complement cadaveric teaching to revisit areas already dissected and for quick revision.

## Introduction

Dissection is indispensable for a correct and comprehensive knowledge of gross anatomy which can translate into safe and efficient clinical practice, but medical institutes worldwide have been facing a paucity of cadavers, with procurement further hindered during the COVID pandemic [1]. With the emphasis on competency-based medical education, regulatory bodies insist on inculcating clinical reasoning skills from the pre-clinical years, which require an understanding of imaging and three-dimensional anatomy [2]. This requires the student to understand pattern and form which requires time and opportunity for reconstruction, testing, validation and modification of the image. Over the years, it has also been observed that students are becoming averse to the cadaver due to increasing awareness of dangers related to the components of the embalming fluid like formaldehyde and also complain of dissection as a stressful instruction [3]. With digitalization having made fast inroads into medical education, the role of dissection has been modified as each student reverts back to an atlas on his smartphone or device. Since defining the exact anatomical site of a lesion is crucial for physicians to diagnose, differentiate and treat safely, adequate anatomical knowledge is essential for surgeons and anyone performing an invasive procedure on a patient [4]. However, with the worldwide curricular reforms those have resulted in a reduction both in the gross anatomy teaching hours and its context, there is a need to do a serious re-examination of the way in which anatomy is taught [5]. Medical institutions in developing countries like India are quickly joining the bandwagon of acquiring virtual dissection tools, but it remains to be scientifically assessed if these tools can be implemented into the curricula and whether they will lead to effective teaching and learning. There is a need to evaluate whether the use of computer simulation techniques to teach gross anatomy to students is an equal or better technique as compared to cadaveric dissection. Thus, the study was undertaken with the aim to evaluate the acceptability, feasibility and effectiveness of the introduction of computer-based simulation for teaching gross anatomy to medical undergraduates. This article was previously presented as a poster at the Experimental Biology 2021 conference held virtually on April 27-30, 2021 and published as a meeting abstract as cited in https://faseb.onlinelibrary.wiley.com/doi/10.1096/fasebj.2021.35.S1.05064.

## Materials and methods

This prospective crossover randomized controlled study was conducted with MBBS first-year students in the Department of Anatomy. All 200 students enrolled (120 males (60%) and 80 (40%) females), with the institute, participated in the study. Sensitization of faculty members was done by discussing with them the proposed plan of study. The students were divided into two groups of hundred students each by a process of simple random sampling. Two broad topics from head and neck anatomy were undertaken: the temporal-infratemporal region and deep dissection of the neck as these are both important regions of head and neck gross anatomy and involve visualization and spatial arrangement of a number of anatomic structures.

The data tool included single correct response, multiple choice questions that tested the ability of students to analyze clinical and spatial anatomy. Questions that involved identification and recall from images were also included. Anonymous Feedback Survey Questionnaires comprising 12 statements to be scored with the aid of a five-point Likert scale and qualitative data was collected in the form of responses to two open-ended questions and delivered online through Google survey forms. Before attempting the feedback questionnaire and as mandated by the directions of the ethics committee, the participants were informed that filling out the survey was voluntary and anonymity would be maintained at all times as no email addresses or names were collected.

The lesson plan and competencies to be covered were decided according to the National Medical Commission of India, Competency Based Medical Education Under Graduate Curriculum-1 [6]. A series of videos were shot while dissecting the cadaver and while teaching on the computer simulation table. These videos were analyzed for any disparity in points covered via the two techniques and serially arranged proceeding from superficial to deeper layers of the body. Didactic lectures were delivered online on the pre-decided topics via the Zoom app. Pre-test was given to all the students via timed Google forms. Pre-test for both sessions had 10 items as multiple-choice questions with one single correct answer. Before giving the pre-test, a mock drill was done a few days before by giving a multiple-choice questions (MCQ) test on these timed Google forms so that students get acclimatized to timed Google tests. The students were then divided into two groups by random allocation. One group was taught gross anatomy on the cadaver by online video projection of dissection videos prepared on the cadaver using Zoom. The second group was instructed on the computer-based simulation table again projected online by Zoom. Since this study was done during the lockdown period where students were being instructed online, one group was taught dissection videos prepared on the cadaver and the second group on videos prepared on a computer simulation table. In both cases, as these were pre-recorded videos, teaching was kept interactive as far as possible and paused when requiring further explanation by the students. The students in both groups were then asked to fill out post-test questionnaires using Google form which were time limited. The post-test items were the same as pre-test questions with the order of appearance scrambled. This teaching methodology was followed for two broad topics of head and neck gross anatomy. Since all the undergraduate medical students enrolled in year 1 were given the teaching intervention, they were invited to provide their anonymous feedback for the same via a Google survey form regarding their perception and satisfaction with the use of both modalities only if they consented to it. The feedback form had 10 items based on a five-point Likert scale and four open-ended questions. It was duly assured that all information shared would be kept confidential and in no way the student’s identity be revealed. It was also assured that the study or information gathered by it will not affect the students teaching and learning opportunities or academic grades. The teachers’ perceptions of the students' understanding of anatomy and the teaching methodology were assessed by written feedback forms. The form had 11 items based on the Likert scale and three open-ended questions.

Since the responses to online pre- and post-tests and feedback forms were on Google survey forms, they were automatically shifted to Excel sheets. Data from only those participants who had given both tests of a particular session were included in the study. Quantitative analysis included proportions and mean. Chi-square was used to compare proportions. Paired sample T-Test was used to compare pre- and post-test scores. The satisfaction index was calculated for Likert scale-based questions in the feedback forms. Themes were identified for the responses to open-ended questions in both faculty and student feedback forms and tabulated. Satisfaction index (S.I.) for all the statements was calculated using the formula: [(n_1_ * 1) + (n_2_ * 2) + (n_4_ *4) + (n_5_ * 5)] * 20 / (n_1_ + n_2_+ n_4_+ n_5_), where n is the total number of students rating the statement under reference. Scores were determined as follows: 1 = strongly disagree, 2 = disagree, 3 = neutral, 4 = agree, and 5 = strongly agree [7].

## Results

The teaching intervention was administered to all 200 students enrolled in the first-year undergraduate program. Out of these 200, a total of 135 and 149 students participated in Session 1 (temporal and infratemporal region gross anatomy) and Session 2 (deep dissection of the neck), respectively, by responding to the online tests (no students were excluded from the teaching intervention, but data was taken from only those who gave both the pre-test and post-test in a given session). Students were administered a pre-test before the teaching session and a post-test after the session. Since some students who gave the pre-test abstained from the post-test, data from the ones who gave both tests were included for analysis. The demographic profile of the subjects is depicted in Table 1.

**Table 1:** Demographic Characteristics of the Participants

	Variable	Pre-test	Post-test	Number of students who attempted both the tests
Session 1: Temporal and infratemporal region	Total	135	139	135
	Male	85 (62.9%)	90 (64.7%)	
	Female	46 (34.1%)	49 (35.3%)	
Session 2: Deep dissection of the neck	Total	169	169	144
	Male	124 (73.4%)	118 (69.8%)	
	Female	45 (25.6%)	51 (30.1%)	

The pre-test score of head and neck (topic 1) among the cadaveric teaching group was 4.90±1.90, while in the computer simulation group, this score was 5.14±1.82. No significant difference was observed in the mean score of both techniques (p=0.467); hence, the subject selection was unbiased for the two techniques. The post-test score among the cadaveric teaching group was 6.63±2.13, while in the computer simulation group, this score was 7.39±2.00. A significant difference was observed in the mean score of both techniques (p=0.033). The mean score of computer simulation teaching was significantly more than the cadaveric teaching technique. A further significant increase from pre- to post-test was found in both cadaveric and computer techniques. However, more change was found in the computer technique as its t-value is more than the cadaveric technique as depicted in Tables 2, 3. The pre- to post-test score difference of Session 1 among the cadaveric technique group was 1.96±1.64, while in the computer technique group, this score difference was 2.18±1.42 as seen in Table 4. No significant difference was observed in the mean score difference between the techniques (p=0.443). The pre- to post-test score difference of Session 2 among the cadaveric technique group was 0.70±1.95, while in the computer technique group, this score difference was 0.97±1.50. No significant difference was observed in the mean score difference between the techniques (p=0.356).

**Table 2:** Comparison of Pre- and Post-Test Scores of Session 2

Session 2	Score				t-value	p-value
	Cadaveric		Computer			
	Mean	SD	Mean	SD		
Pre-test	6.14	2.09	6.58	2.02	-1.37	0.172
Post-test	6.67	1.96	7.23	1.83	-1.73	0.086
Significance	t=2.90, p=0.005		t=5.45, p<0.001			

**Table 3:** Comparison of Pre- and Post-Test Score Difference of Trainings

Topic	Score difference: pre- and post-test				t-value	p-value
	Cadaveric		Computer			
	Mean	SD	Mean	SD		
Topic 1	1.96	1.64	2.18	1.42	-0.77	0.443
Topic 2	0.70	1.95	0.97	1.50	-0.93	0.356

A further significant increase from pre- to post-test was found in both cadaveric and computer techniques. However, more change was found in the computer technique as its t-value is more than the cadaveric technique as shown in Table 4.

**Table 4:** Analysis of Student Feedback on the Teaching Techniques Percentages are given in brackets.

		Computer simulation teaching					Cadaveric teaching				
	Variable	Strongly disagree	Disagree	Not sure	Agree	Strongly agree	Strongly disagree	Disagree	Not sure	Agree	Strongly agree
1.	Helped in understanding 3D anatomy	1 (1.3%)	7 (9.3%)	8 (10.7%)	39 (52%)	20 (26.7%)	1 (1.3%)	17 (22.4%)	24 (31.6%)	26 (34.2%)	8 (10.5%)
2.	Helped in understanding relations of body structures	1 (1.3%)	2 (2.7%)	9 (12%)	45 (60%)	18 (24%)	3 (3.9%)	15 (19.7%)	21 (27.6%)	33 (43.4%)	4 (5.3%)
3.	Increased capacity to grasp surface anatomy	2 (2.7%)	6 (8%)	13 (17.3%)	42 (56%)	12 (16%)	0 (0%)	14 (18.7%)	28 (37.3%)	30 (40%)	3 (4%)
4.	Should be the first method for teaching	4 (5.3%)	15 (20%)	18 (24%)	27 (36%)	11 (14.7%)	9 (11.8%)	28 (36.8%)	14 (18.4%)	22 (28.9%)	3 (3.9%)
5.	This method could help in quick revision	2 (2.7%)	9 (12%)	13 (17.3%)	34 (45.3%)	17 (22.7%)	1 (1.3%)	18 (23.7%)	25 (32.9%)	28 (26.8%)	4 (5.3%)
6.	Can improve retention and recall	1 (1.3%)	2 (2.7%)	17 (22.7%)	44 (58.7%)	11 (14.7%)	0 (0%)	8 (10.6%)	25 (32.9%)	38 (50%)	5 (6.6%)
7.	Could enhance performance in exams	2 (2.7%)	2 (2.7%)	19 (25.3%)	42 (56%)	10 (13.3%)	0 (0%)	10 (13.3%)	26 (34.7%)	36 (48%)	3 (4%)
8.	Safer method in pandemic	1 (1.3%)	2 (2.7%)	6 (8%)	32 (42.7%)	34 (45.3%)	2 (2.6%)	6 (7.9%)	15 (19.7%)	31 (40.8%)	22 (28.9%)
9.	Dissection was aligned with the lecture	1 (1.4%)	1 (1.4%)	12 (16.2%)	45 (60.8%)	15 (20.3%)	1 (1.3%)	5 (6.7%)	11 (14.7%)	43 (57.3%)	15 (20%)
10.	Sessions were well-planned	0 (0%)	1 (1.3%)	9 (12%)	38 (50.7%)	27 (36%)	0 (0%)	4 (5.3%)	13 (17.1%)	42 (55.3%)	17 (22.4%)

The pre- to post-test score difference of Topic 1 among the cadaveric technique group was 1.96±1.64, while in the computer technique group, this score difference was 2.18±1.42 as seen in Table 4. No significant difference was observed in the mean score difference between the techniques (p=0.443). The pre- to post-test score difference of Topic 2 among the cadaveric technique group was 0.70±1.95, while in the computer technique group, this score difference was 0.97±1.50. No significant difference was observed in the mean score difference between the techniques (p=0.356).

A total of 151 students gave feedback out of which 108 (71.5%) were males and 43 (28.5%) were females. As shown in Table 5, the computer simulation technique had more responses of agree and strongly agree for understanding 3D anatomy, relations of body structures and grasping surface anatomy. There were more responses of agree and strongly agree choices for the computer simulation technique to be the first method of teaching and that could help with quick revision and recall, and thus enhance performance in exams. More number of participants strongly agreed that teaching on the computer simulation table would be safer in the pandemic. Both groups strongly agreed that the sessions were well-planned and aligned with the lectures.

**Table 5:** Student Feedback Satisfaction Index for the Cadaveric and Computer Simulation Techniques The range of satisfaction index is 1-100 [7].

S.No.	The teaching method	Cadaveric teaching	Computer simulation
1.	Gave me a good insight into 3D understanding of the human body	66.05	78.67
2.	Increased my understanding of the relations of body structures and organs	65.26	80.53
3.	Increased my capacity to grasp surface anatomy	65.87	74.93
4.	This technique should be the first method of teaching	55.26	66.93
5.	Can be helpful in quick revision for examination preparation	64.21	74.67
6.	Improved my retention and recall of gross anatomy	70.53	76.53
7.	Will enhance my performance in theory examinations	68.53	74.93
8.	Teaching sessions were well-planned	78.95	84.27
9.	Were well aligned with the gross anatomy lectures	77.60	79.46
10.	Considering physical distancing due to COVID pandemic, this learning method can be used safely in the future	77.11	85.60
	Average score	68.94	77.65

The satisfaction index of the computer simulation technique was greater than the cadaveric technique for all variables noted and in summation, as shown in Table 6. The faculty also showed a better acceptability of the computer simulation teaching technique. The general consensus was that although it can provide opportunities to students for revision, avenues for more interaction and student-led dissection, it can never replace cadaveric dissection.

**Table 6:** Thematic Response of Faculty Feedback A total of seven faculty provided the feedback.

Questions	Computer simulation	Cadaveric
Aspects which you liked best	Useful for both online and offline teaching. Provides clear understanding. Boon during COVID time; helps to cover many topics in small time quick revision; can help to make concepts; explanation was very comprehensive; clear visualization to all students at one go; can dissect same body again and again; less time-consuming; and co-relate cross-sectional anatomy	Can never replace cadaveric teaching
Aspects which you did not like	Internet connectivity with online teaching; in online teaching, lag between speaker’s talk and demonstration; students are unable to feel and dissect structures themselves; and technical part of computer simulation is difficult	Orientation of anatomical region little difficult, Cannot get the feel of structures
Suggestions for improving	Provide videos to students for revision; plan for more interaction; a session every week/biweekly to clear doubts; provide videos to students for revision; and allow students to use the simulation table themselves	For thorough concepts, need to add both cadaveric and computer simulation. Dissection involving big structures/muscles by cadaveric and minute dissections by computer simulation

## Discussion

Anatomy teaching should prepare students for clinical practice, by providing them with a 3D perspective of anatomical structures, using the whole armamentarium of tools provided by modern technology along with the irreplaceable cadaveric teaching. Although simulation in medicine has been in vogue since the ninth century when Madame du Coudray used mannequin pelvis and babies to train midwives for childbirth, its use became more rampant with the introduction of versatile human simulators by the late 1990s and early 2000s [8]. Simulation has been used in training for departments of Anesthesia, Pharmacology, Physiology, Surgery and Pediatrics to name a few [9] for quite some time, but their use in anatomy to replace cadaveric dissection has been quite debatable for quite some years now. If we consider the requirements according to the Competency Based Medical Education (CBME) curriculum rolled out in 2019, the integration and the emphasis on a need-based schedule rather than time-based curricula based on cadaveric dissection do not easily facilitate vertical integration. The fixed dissection sequence limits the ability of cadaveric dissection to integrate into case-based curricula. It is difficult to personalize as students are unable to correct dissection mistakes or re-visit completed dissections [4]. Many medical schools have difficulty in the procurement of cadavers, yet medical student numbers are constantly increasing, along with awareness about the harmful effects of formalin. This coupled with greater emphasis on clinically relevant anatomy enhances the role and scope of virtual or digital dissection, not just for anatomists, but also for all medical educators like radiologists and surgeons [10]. Students over the years are also getting averse to cadaveric dissections because of the limitations like smell, color, inability to change the position or auscultate [11]. Another very valid point emerging is that the concept of spatial anatomy needed for a physician to understand radiographs or a surgeon for interventions is difficult to learn in physical cadaver dissections, which only promotes recall of lexical information [12]. To counter these drawbacks, virtual dissection or computer simulation dissection is beneficial as it works on the principle of manipulating computed tomography (CT) scan data in three dimensions to reveal the different organ systems and their anatomical relationships [4].

With the new interest in virtual or computer simulation dissection, it became important to document its effectiveness as compared to the traditional methods. Zhao et al. [13] in 2020 published a meta-analysis of randomized controlled trials and found that virtual dissection significantly increased learners’ examination scores compared with traditional learning as seen in our study. Of the 15 studies that met their inclusion criterion, only two compared virtual dissection with cadaveric dissection that reiterates our aim to undertake this study to compare the pedagogical benefits of computer simulation with cadaveric dissection.

Hariri et al. [14] compared two groups of medical students who studied shoulder joint anatomy using either a second-generation virtual reality surgical simulator or images from a textbook. The mean scores the students obtained out of 7 were 3.1±1.3 for the simulator group and 2.9±1.5 for the textbook group (p=0.70). Although our study compares computer simulation teaching with cadaveric teaching, we too found that in both our teaching sessions, the mean post-test score of the computer simulation group (7.39 and 7.23) was better than the cadaveric group (6.63 and 6.67), respectively. A similar study by Prinz et al. [15] compared surgical videos with 3D computer animations to teach ophthalmology procedures to medical students resulting in a significant improvement in knowledge and a better acceptance of multimedia-assisted teaching.

Our study showed that the satisfaction index of the computer simulation technique (77.65) was greater than the cadaveric technique (68.94), which is in concordance with the findings of Keedy et al. [16], who reported higher overall satisfaction and ease of use with the 3D computer teaching module for teaching hepatobiliary anatomy. The study by Hariri et al. [14] also reported that students felt greater ease of use with the surgical simulator.

Gould et al. [17] tested the usability and acceptability of a computer-assisted tool to teach neuroanatomy and found that 85.7% of students agreed it could improve their performance in an exam, whereas in our study, 56% study agreed and 13.3% strongly agreed that computer simulation technique would help in quick revision and performance before an exam as compared to 48% and 4% by cadaveric technique. While 50.7% of our students agreed that computer simulation techniques could be the first method of teaching, only 31.6% of their respondents said that the computer-assisted technique could remain as a stand-alone plan, although 97.5% of them agreed that it could supplement other traditional learning methods. In comparison, only 88% of the faculty responded favorably to our faculty feedback of 80% to the computer simulation teaching.

A study done by Saltarelli et al. showed that teaching done in the cadaveric laboratory showed a significant advantage over multimedia simulation programs [18]. This raises concerns that the incorporation of simulation into the curriculum requires careful alignment between learning objectives and competencies. Much of the feedback we received confirmed that students observed that “The topics covered are correlated to each other”, gave them “better 3D orientation and better understanding”, “gave the exact location of structures in human body”, “can be used for revision purpose”, “it enhanced my practical knowledge of human body”, “it was understandable” and “It was related to the topic taught”.

Anatomy education should aim to continue in an integrated manner throughout medical teaching and learning. The aim of Competency-Based Medical Education is to produce lifelong learners who can understand spatial and radiological imaging and analyze it to the disease condition seen in the living anatomy, which is much more doable with computer-based simulation anatomy. Unfortunately, technology has its limitations too, like the lack of haptics which is achieved by incising the skin and touching the structures. Also, the cost of obtaining and maintaining these tools is a big limiting factor, if added to the cost of training the facilitators, and then becomes a major challenge for developing countries [19]. Still, institutions that can afford these expensive tools need validation by studies like the present one and that by Wish-Baratz et al. [20] to document the feasibility and effectiveness of this teaching tool.

Limitations

This study was done for only two broad topics of head and neck gross anatomy but, for a better translation, should cover limb and visceral anatomy too to give a better understanding of which teaching method would be acceptable, effective and feasible for teaching and learning gross anatomy of the abdomen, pelvis and thoracic viscera and the extremities.

## Conclusions

The study concludes that it is feasible to use computer simulation techniques for teaching anatomy to medical undergraduates. Also, it was considered to be an effective method of learning by the students as it was found to induce a significant improvement in their understanding and perception. Teaching faculty also accepted the computer simulation technique as an effective method of teaching as computer-based simulation teaching provides for quick revision, can help to make concepts by providing clear visualization to all students at one go and students can dissect the same region again and again, but it was agreed that for thorough conceptualization, students need to be exposed to both cadaveric and computer simulation techniques for gross anatomy teaching as the haptic feedback obtained from cadaveric dissection is unparalleled. Potential applications and implications can be useful for undergraduate and postgraduate students who wish to revisit anatomy during surgical training years, for time-restricted curricula and as a combination tool with other instructional methods available.
